# Enhancement of Antibacterial Activity of Orange Oil in Pectin Thin Film by Microemulsion

**DOI:** 10.3390/nano8070545

**Published:** 2018-07-19

**Authors:** Pensak Jantrawut, Kasidech Boonsermsukcharoen, Kanyanut Thipnan, Tanpong Chaiwarit, Kyu-Mok Hwang, Eun-Seok Park

**Affiliations:** 1 Department of Pharmaceutical Sciences, Faculty of Pharmacy, Chiang Mai University, Chiang Mai 50200, Thailand; jjkasidech@gmail.com (K.B.); kanyanut.thip@gmail.com (K.T.); tannahupp@gmail.com (T.C.); 2 School of Pharmacy, Sungkyunkwan University, Suwon, Gyeonggi-do 440-746, Korea; bemseotj@gmail.com (K.-M.H.); espark@skku.edu (E.S.P.)

**Keywords:** microemulsion, orange oil, pectin, thin film, antimicrobial activity

## Abstract

The purpose of this study was to prepare orange oil microemulsion (ME) and to investigate the antimicrobial activity of film containing orange oil ME. First, surfactants and co-surfactants were screened on their efficiency to form ME using pseudo-ternary phase diagrams. The influences of surfactant and co-surfactant mass ratios were studied and optimized ME-loaded-films were prepared. Then, films containing orange oil ME were characterized by SEM and texture analyzer, and then evaluated for antimicrobial activity against *Staphylococcus aureus* and *Propionibacterium acnes* using an agar disc diffusion method. The results showed that Tween 80 as surfactant and propylene glycol as co-surfactant at a 1:1 ratio possessed the maximum ME area. Three ME formulations of ME 20, ME 25, and ME 30, which consisted of 20, 25, and 30% *w*/*v* of orange oil were prepared, respectively. All ME formulations showed particle sizes of about 60.26–80.00 nm, with broad a polydispersity index of 0.42. The orange oil ME films exhibited higher elastic values than the control. The diameters of inhibition zones for FME 20, FME 25, and FME 30 against *P. acnes* were 13.64, 15.18, and 16.10 mm, respectively. Only the FME 30 had an antimicrobial activity against *S. aureus* with 8.32 mm of inhibition zone. Contrarily, the control film had no antimicrobial activity against both bacteria. In conclusion, the present study found that the antibacterial activity of orange oil in pectin thin film could be enhanced by preparing orange oil as an ME before loading into pectin thin film.

## 1. Introduction

Natural aromatic compounds and flavors such as fruit and vegetable essential oils are extensively used in food, pharmaceutical, cosmetic products, and perfumes [1,2,3]. Orange oil is one of the most useful and beneficial essential oils. The popularity of orange oil is due to its pleasant aromatic scent, as well as therapeutic properties such as anti-inflammatory, antiseptic, anti-depressant, tonic, carminative, antispasmodic, diuretic, and as a sedative [3,4]. The antimicrobial activity of the orange oil was also evaluated and it exhibited significant inhibitory effects against many bacteria such as *Staphyloccocus aureus, Enteroccocus feacalis*, *Klebsiella pneumoniae*, *Proteus vulgaris*, *Pseudomonas aeruginosa, Escherichia. Coli*, and *Candida albicans* [5,6]. Limonene, which is the most abundant compound in orange oil, has been implicated to be an active ingredient for antimicrobial effect [7].

Nowadays, hydrocolloid bandages, which are flexible bandages made of a water-attracting material attached to a thin plastic or natural film, has become more popular, particularly for acne. The thin plastic film is usually made from polyurethane, whereas the natural ones are usually made of carboxymethyl cellulose, gelatin, and pectin, which are the polymers that have lots of water-binding groups in their chemical structure. Natural polymer like pectin is an interesting candidate for thin film dosage form, because pectin is comparatively cheap, abundant, non-irritating, and non-toxic [8]. In our preliminary study, orange oil was loaded into pectin thin film and tested for antibacterial activity against *S. aureus* and *Propionibacterium acnes*, which are common skin infection microorganisms, but its application has been associated with problems such as lack of solubility in hydrophilic pectin solution, volatility during film formation processing resulting in low limonene loading content, and failure to inhibit any of the tested strains. Therefore, finding methods to increase the solubility and biological activity focusing on the antibacterial activity of orange oil in hydrophilic film formulation is very important, being a turning point in the use of aromatic compounds, especially essential oils in film dosage form. In this regard, emulsion technology, especially the microemulsion (ME) technique, seems to be one of the most promising processes to solve these problems.

Microemulsion has generated considerable interest over the years as potential drug delivery systems [9,10]. One of the most important applications of MEs is to incorporate lipophilic active ingredients into aqueous-based formulations [11]. Moreover, MEs can easily be fabricated from active ingredients using simple processing operations, such as mixing and shearing. Microemulsions are thermodynamically stable systems that typically consist of oil, surfactant (sometimes co-surfactant/co-solvent), and water [12,13,14], although orange oil MEs and nanoemulsions have been developed by some researchers [15,16,17]. However, there are limited approaches for using orange oil ME to enhance antibacterial activity in thin film for pharmaceutical applications. Thus, the focus of our current study was established to develop orange oil in an ME system, and then incorporate it into pectin thin film and investigate the effect of film formulation loaded with orange oil ME on film morphology and mechanical property, limonene loading content, as well as antibacterial activity.

## 2. Materials and Methods

### 2.1. Materials

Non-amidated low methoxy pectin (LMP; Unipectine OF300C; DE = 30%) was purchased from CargillTM (Saint Germain, France). Orange oil was purchased from Thai-China flavors and Fragrance Industry Co., Ltd. (Nonthaburi, Thailand). Ammonium molybdate was purchased from Sigma Chemicals, St. Louis, MO, USA. Calcium chloride (CaCl_2_) was purchased from Merck (Damstadt, Germany). Mueller Hinton agar (MHA) were purchased from Becton Dickinson (Nebraska, NE, USA). Tryptic soy broth (TSB) was obtained from HiMedia Laboratories Pty. Ltd. (Mumbai, India). *Staphylococcus aureus* (ATCC 25923) and *P*. *acnes* (ATCC 6919) were obtained from the BIOTEC Culture Collection, National Center for Genetic Engineering and Biotechnology (Manassas, VA, USA). Distilled water served as the solvent for preparing ME and film solutions. All reagents were of analytical grade.

### 2.2. Preparation and Optimization of Orange Oil ME Formulations

#### 2.2.1. Screening and Selection of Surfactants

Three different surfactants namely, Tween 20, Tween 40, and Tween 80 were screened. The solubilization capacity of surfactants was studied using 3 mL of 15% *w*/*v* aqueous solution of each surfactant to which aliquots of 1 µL of orange oil was added with vigorous vortexing until the solution became cloudy [18]. Also, emulsification ability of the above-mentioned surfactants was screened. Fifty mg of surfactant was added to 50 mg of orange oil. This isotropic mixture, 100 mg, was diluted with distilled water (25 times) to yield a fine emulsion. The emulsion were allowed to stand for 2 h to note for any change in turbidity, and its transmittance was assessed at 650 nm by UV spectrophotometer (UV-2450, Shimadzu, Japan) using distilled water as blank [19].

#### 2.2.2. Screening and Selection of Co-Surfactants

The selection of co-surfactants was done on the basis of ME region. Tween 40 or 80 was mixed with three types of co-surfactants, namely, glycerin, propylene glycol, and polyethylene glycol. S_mix_ ratio (1:1) was kept constant. The phase diagrams of ternary systems (oil, surfactant, and aqueous phases) were constructed using aqueous titration or spontaneous emulsification method [20]. Ten combinations in different weight ratios of orange oil and S_mix_ (10:0, 9:1, 8:2, 7:3, 6:4, 5:5, 4:6, 3:7, 2:8, 1:9, and 0:10) were prepared in glass vials at room temperature. Each ratio of S_mix_ and oil phase was then titrated drop-by-drop continuously with water using micropipette by vortex mixing till it turned turbid. The phase behavior of each ternary system during titration was observed minutely. The percentage composition of the component in each ternary system was determined and the observed results were plotted on triangular co-ordinates to construct the phase diagrams.

#### 2.2.3. Influence of Surfactant and Co-Surfactant Mass Ratio on ME Formation

The selected surfactant and co-surfactant (S_mix_) were blended at the weight ratios of 1:1, 1:2, 2:1, 1:3, and 3:1. Different combinations in different weight ratios of oil and S_mix_, 10:0, 9:1, 8:2, 7:3, 6:4, 5:5, 4:6, 3:7, 2:8, 1:9 and 0:10, were taken. Aqueous titration method was employed for the construction of the pseudo-ternary phase diagrams. Subsequently, the mixtures were evaluated visually and the ME phase was identified as the region in the phase diagram where clear, easily flowable, and transparent formulations were obtained.

#### 2.2.4. Preparation of ME Formulation

Orange oil ME was prepared by adding 20, 25, and 30% *w*/*v* orange oil in different S_mix_ (Tween 80 and propylene glycol) ratios which were 70, 65, and 60%, respectively (Table 1). Then the mixture was mixed with the aid of a vortex mixer and made up to 100% with the slow addition of distilled water with continuous stirring.

#### 2.2.5. Morphology and Vesicle Size Determination of ME

A drop of ME was applied on a 300-mesh formvar carbon-coated copper grid (FCF400-Cu-50, Electron Microscopy Sciences, Hatfield, UK) on paraffin, and the sample was allowed to adhere on the formvar for 10 min. The remaining ME was removed and a drop of 2% aqueous solution of ammonium molybdate was applied for 5 min. The remaining solution was then removed. The sample was air dried and examined with a transmission electron microscope (JEM-2200FS JEOL, JEOL Ltd., Peabody, MA, USA). The morphology of the particle was observed. The particle sizes of orange oil MEs were determined using dynamic light scattering (DLS), Zetasizer 300HSA (Malvern Instruments, Malvern, WR14 1XZ, UK), based on photon correlation spectroscopy. Analysis (*n* = 3) was carried out for 100 s at room temperature (25 ± 2 °C). All samples were performed without any diluent for the particle size measurement.

### 2.3. Preparation and Characterization of Orange Oil ME-Loaded Pectin Thin Film 

#### 2.3.1. Film Preparation

The films were prepared using modified ionotropic gelation with solution-casting techniques as described in a previous study [21]. In this study, each ME formulation, at fixed 3% *w*/*v* of orange oil, was added into the pectin solution (Table 1). Then, each formulation (5 mL) was casted on a dialysis membrane. The casted solution on dialysis membrane was placed on 3% CaCl_2_, which was supported by a plastic box, for 5 min. The gelled film was formed and transferred to a plastic plate, then dried in a hot air oven at 30 °C for 48 h. After that, dried film was peeled and kept in a desiccator for further experiments. Control film, which was used for comparing to ME-loaded pectin thin films, were made by adding 3% *w*/*v* of orange to the pectin solution, and following the aforementioned preparation.

The minimum inhibitory concentration (MIC), which is the lowest concentration of a chemical which prevents the growth of a bacterium, was used for selecting percentage of orange oil loading in the film. In this study, the MICs of orange oil against the growth of *S*. *aureus* and *P*. *acnes* were at the concentration of 2.5 mg/mL. Therefore, the concentration of orange oil (3%) above MIC was then selected and used for film preparation.

#### 2.3.2. Morphological Characterization of the Films

The morphology was examined using scanning electron microscopy (SEM) with a JEOL scanning electron microscope (JSM-5410LV, JEOL Ltd., Peabody, MA, USA) at 10 kV under low vacuum mode. The film characterizations were performed without any coating solution at magnifications of 150×. The thickness and surface of films were evaluated.

#### 2.3.3. Film Thickness

The thickness of each 3 × 3 cm^2^ sized film was measured at 10 points using a thickness gauge (GT-313-A, Gotech Testing Machines Inc., Taichung, Taiwan). The mean film thickness (in mm) with the standard deviation was calculated.

#### 2.3.4. Testing of Tensile Properties

The dried film was cut into a rectangular shape (2 cm × 7 cm). The tensile properties of the films were tested using a texture analyzer TA.XT Plus (Stable Micro Systems, Surrey, UK). The film sample was clamped between two grips and the initial gauge length was set at 5 cm. The film was pulled using a crosshead speed of 1 mm/s and the load cell was set at 100 N. At the point of breaking, force (N) and elongation (mm) were recorded. Only films that broke at the center of the strip were used for data analysis. Each experiment was repeated six times. The tensile properties of the films were characterized by the tensile strength, percent elongation, and Young’s modulus [21].

#### 2.3.5. Limonene Loading Content in Pectin Thin Film

The limonene loading content in the films was determined using GC-MS (Agilent-Technologies 6890N network gas chromatographic system combined with Agilent-Technologies 5973 inert XL mass selective detector and Agilent-Technologies 7683B auto-injector, Little Falls, CA, USA). The films were cut into a 50 mm^2^ area and their weights were recorded. They were then cut into small pieces. Limonene was extracted from the films by soaking them in 5 mL ethanol for 24 h. The extracted solution was diluted to 100 µg/mL (theoretical concentration was calculated by the weight of each film). Each sample was analyzed in triplicate. Limonene content was determined from the standard curve of limonene in absolute ethanol, which was linear with a high correlation coefficient (*r*^2^ = 0.9988). The following regression equation was obtained: *y* = 169867*x* − 397775, where *y* is the peak area and *x* is the concentration of limonene (mg/L). The limonene loading was calculated using the following equation:

(1) $$\mathrm{Limonene}\;\mathrm{loading}\;\mathrm{content}\;(\%)=\frac{\mathrm{Aq}\;\left(g\right)}{C\;\left(\%\frac{w}{w}\right)\;\times \;W\;\left(g\right)}\;\times \;100$$

where Aq is the actual quantity of limonene present in the film (g), C is the concentration of limonene (%w/w), and W is the film weight (g).

### 2.4. Antibacterial Activity of Orange Oil ME-Loaded Pectin Thin Film

In vitro antibacterial activity of orange oil ME-loaded pectin thin film was determined using the agar disc diffusion method which was applied using Kirby-Bauer antibiotic testing [22,23]. *Staphylococcus aureus* and *P. acne* were activated in TSB and incubated for 6–10 h. Absorbance was then measured at 600 nm. The activated bacterial turbidity that equals 10^8^ cfu/mL was used and cultivated on MHA plates. The orange oil ME-loaded pectin thin films (4 mm diameter) were put on the MHA plates. Five serial concentrations of orange oil (0.125 to 150 mg/mL) were prepared. Then, they were sterilized using 0.2 μm membrane filtration method, dropped on sterile discs, and put on the plate in order to determine MIC. Ampicillin was used as a positive control. One microliter of Tween 80 and propylene glycol (which was equivalent to the amount in ME-loaded film) were also investigated for their antibacterial activity. After that, the MHA plates were incubated at 37 °C for 48 h, and the diameter of the clear zone was measured using a Vernier caliper. Each experiment was performed in triplicate.

### 2.5. Statistical Analysis

All data are presented as mean ± SD. Kruskal-Wallis test was used to evaluate the significance of differences at *p*-value < 0.05. Statistical analysis was performed using SPSS software version 17.0 (SPSS Inc., Chicago, IL, USA).

## 3. Results and Discussion

### 3.1. ME Preparation and Characterization

#### 3.1.1. ME Preparation

**Figure 1:**
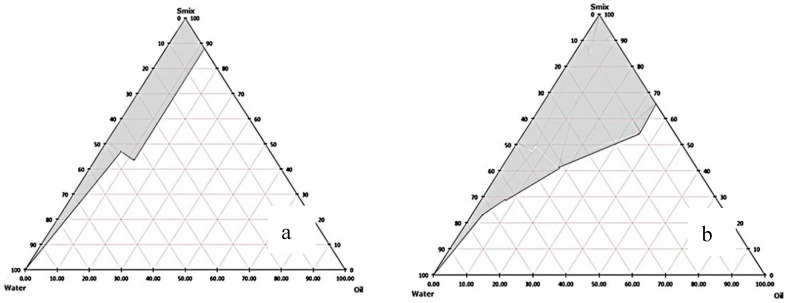
Pseudo-ternary phase diagrams indicating microemulsion (ME) region (grey area) comprising of orange oil and different S_mix_: (**a**) Tween 40 and propylene glycol, and (**b**) Tween 80 and propylene glycol at 1:1 ratio.

**Figure 2:**
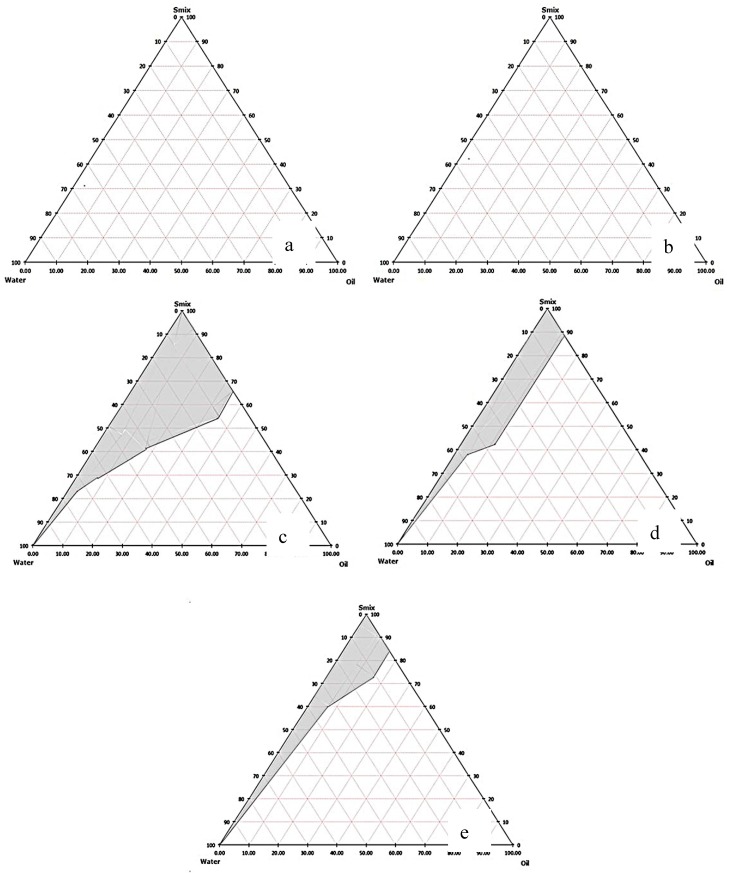
Pseudo-ternary phase diagrams of orange oil and S_mix_ (Tween 80 and propylene glycol) at 3:1 (**a**), 2:1 (**b**), 1:1 (**c**), 1:2 (**d**), and 1:3 (**e**) ratios. Grey area represents clear isotropic ME.

The solubilization capacity of surfactants was studied by aliquot of orange oil to aqueous solution of each surfactant until the solution became cloudy. The same maximum volume of orange oil solubilized in Tween 20, 40, and 80 aqueous solutions was found at 3 mL. The emulsification ability of the surfactant in terms of percent transmittance was Tween 40 (93.93%)~Tween 80 (92.93%) > Tween 20 (76.73%). In general, the lipophilic chains of surfactant should be short or at least contain a fluidizing group such as double bonds in order to allow oil uptake [24] such as Tween 80, which contains a double bond in its lipophilic chain. However, in this present study, there was no significant difference between percent transmittance of Tween 40 and 80. Therefore, they were selected as surfactants for further co-surfactant screening. The emulsification capability of co-surfactants was determined by ME region in the pseudo-ternary phase diagrams. These were compared at a fixed S_mix_ (1:1). The orange oil ME regions were observed only when propylene glycol was used as a co-surfactant in both Tween 40 and 80 containing systems (Figure 1). The larger ME area in case of propylene glycol as a co-surfactant was in the Tween 80 system (Figure 1b). Besides, the influence of surfactant and co-surfactant mass ratio on ME formation was assessed. Pseudo-ternary phase diagrams were constructed using orange oil, Tween 80 as surfactant and propylene glycol as co-surfactant (Figure 2). A large ME area was obtained toward the surfactant: co-surfactant at 1:1 ratio (Figure 2c). When the co-surfactant concentration was doubled or tripled (Figure 2d,e), the total area of ME decreased compared to 1:1 S_mix_ ratio. Higher concentration of co-surfactant appeared to have a destabilizing effect on the formation of ME resulting in the reduction of ME area [25]. In addition, when surfactant concentration of S_mix_ was increased (Figure 2a,b), no ME area was observed. It might be due to insufficient co-surfactant concentration, required to reduce the interfacial tension between oil and water [25]. Generally, surfactant helps in the reduction of the interfacial tension by forming a film at the oil-water interface resulting in the spontaneous formation of ME, while the presence of co-surfactants decreases the bending stress of the interface and allows the oil-water interfacial film sufficient flexibility to take up different curvatures required to form ME over a wide range of compositions [26,27]. This result is similar to the result in an earlier study, where the S_mix_ ratio of 1:1 possesses the maximum ME area [28]. Therefore, Tween 80 as the surfactant and propylene glycol as the co-surfactant at 1:1 ratio were selected to prepare orange oil ME in this study. After that, three ME formulations including ME 20, ME 25, and ME 30 were prepared for further experiments (Table 1).

#### 3.1.2. ME Characterization

**Figure 3:**
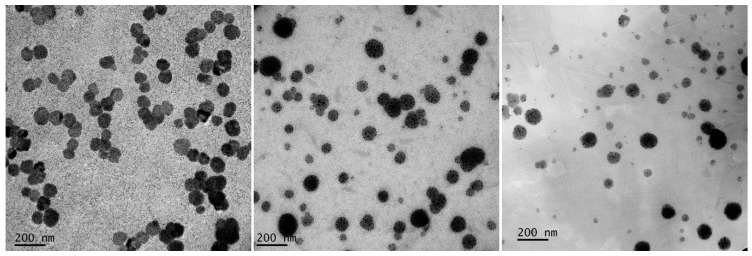
TEM images of orange oil ME: ME 20 (**left**); ME 25 (**middle**), and ME 30 (**right**) at magnification 35,000×.

The characterization of ME was carried out on the basis of percentage transmittance, droplet size, and polydispersity index (PdI), and the results are tabulated in Table 1. Transmission electron microscopy is one of several techniques used to measure the size of ME droplets. In this study, all the optimized orange oil MEs possessed a spherical shape with a size ranging from 50 nm to 100 nm (Figure 3). From DLS measurements, the droplet sizes of ME 20, 25, 30 were 73.42 ± 1.60, 75.17 ± 7.27, and 77.63 ± 0.57 nm, respectively. There was no significant difference (*p* > 0.05) in droplet size on surfactant concentrations in this study. The PdI values of all ME formulations were rather high, indicating non-uniformity of droplet size within each formulation. This may be due to low energy of ME production with the stirring method. In order to produce ME with a high uniformity and proper size of droplets in the dispersed phase, high-pressure homogenizers, high-shear mixers, ultrasonic devices, and membrane techniques are used [29,30]. However, we found that there is no statistically significant difference between the droplet size and PdI values among ME formulations (*p* > 0.05). The zeta potential of the optimized ME was not determined in this study as the stability of ME and lipid emulsions containing non-ionic surfactants does not depend on zeta potential [31].

### 3.2. Film Characterization

#### 3.2.1. Thin Film Morphology

**Figure 4:**
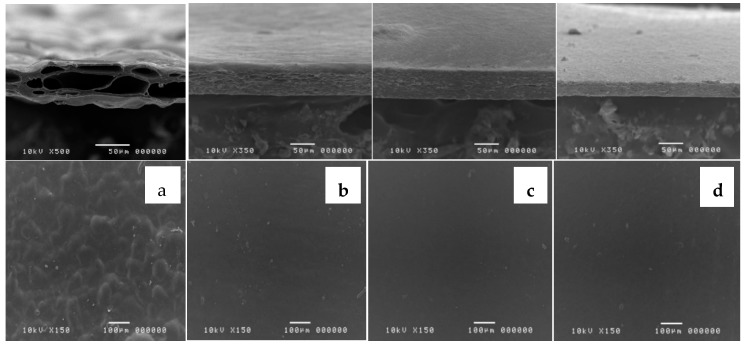
Scanning electron microscopy of the surface (**top**) and cross-section (**down**) of orange oil-loaded film; control (**a**), orange oil ME-loaded films namely FME 20 (**b**), FME 25 (**c**), and FME 30 (**d**).

All orange oil and orange oil ME-loaded films were slightly yellowish-translucent with orange flavor. As seen in Figure 4, the large-size micropores inside the film’s matrix were found when orange oil was incorporated. These micropores occurred when 3% orange oil evaporated from the polymer matrix during the drying process. Our results are consistent with another study which loaded oregano essential oil in quince seed film and also showed a loose structure with micropores inside the film’s matrix [32]. This result was also similar to the findings in a previous study by Ferreira et al. [33] who found that film structure discontinuities were induced by incorporation of wax or oil into the polysaccharide matrix. Interestingly, the current study found that the micropores were reduced by loading orange oil ME into pectin thin film. FME 20, 25, and 30 films showed smooth surfaces and dense film matrices with fewer micropores (Figure 1b–d). This study indicated that ME technology can effectively wrap lipophilic components and lead to a reduction in the volatility of orange essential oil.

#### 3.2.2. Mechanical Properties

The effect of ME loading on the mechanical properties of film containing orange oil ME is of interest. The tensile strength, percent elongation, and Young’s modulus values of orange oil ME-loaded films are shown in Table 2. There was no statistically significant difference of tensile strength, percent elongation, and Young’s modulus values among FME 20, 25, and 30 films. Tensile strength and Young’s modulus of orange oil ME-loaded films significantly decreased, while percent elongation significantly increased compared with the control film. Essential oils have been widely reported to weaken the intermolecular interactions between polymeric chains, resulting in less rigid and more extensible and flexible films [34,35,36]. Our findings were comparable with Otoni et al. [37], who also noticed that reduction in droplet size led to an even more remarkable plasticizing effect, because smaller droplets significantly increased elongation at break and reduced elastic modulus of films.

#### 3.2.3. Limonene Loading Content

Gas chromatography-mass spectrometry analysis showed that the orange oil was comprised of 19 known compounds, and the major compound of this orange oil was limonene (84.57%). Therefore, limonene was used as the marker for loading content study. The limonene loading content of orange oil ME-loaded films is shown in Table 2. All orange oil ME-loaded films exhibited significantly higher limonene loading contents (83–85%) than control film (60.75%). This result is consistent with a morphological study of films. The texture of the orange oil incorporated film turned out to play an important role because micropores indicated the evaporation of orange oil from the polymer matrix, as shown in the loading content results. Films with a fewer and smaller-sized micropores can encapsulate more content of orange oil. On the other hand, films with a larger amount and larger size of micropores show the lowest orange oil content. Moreover, the increase in loading orange oil content could be attributed to enhanced solubility of orange oil in ME. Hence, the increased loading capacity of films to contain more orange oil has been accomplished in this present study. 

### 3.3. Antibacterial Activity of Orange Oil ME-Loaded Films 

The results of the antimicrobial activity of the films against the growth of *S. aureus* and *P. acnes* using agar disc diffusion method are shown in Table 3. Control film, which was the film containing orange oil instead of orange oil ME, failed to inhibit any of the tested strains. Whereas films with incorporated orange oil ME showed the inhibition zones against both bacteria. This might be due to orange oil in control film not being able to release from the film. All orange oil ME-loaded films exhibited an inhibitory clear zone against the growth of *P. acnes*, which was FME 30 (16.10 mm)~FME 25 (15.18 mm) > FME 20 (13.64 mm). Only FME 30 film, however, showed an inhibitory clear zone (8.32 mm) against the growth of *S. aureus*. The small size of essential oil droplets in microemulsion systems ensures a close and wide contact to the surface of the bacteria cell walls and may increase the amount of the essential oil penetrated into the cell [38]. Weiss et al. [39] confirmed that the penetration of antimicrobial compounds in the bacterial cell is higher with reduction in the oil droplet size. Subsequently, the greater amount of orange oil released from microemulsion may increase the bioavailability of the essential oil, and therefore, improve its antibacterial effect. In addition, the thinner film (i.e., FME 30) tended to present more antibacterial activity than thicker ones (i.e., FME 20 and 25). As the differences in droplet size between the ME formulations were not significant, the effect of antibacterial activity of orange oil ME-loaded film in this study is associated with film porosity, film thickness, and the relative amount of the active ingredient. Since each ME-loaded film had identical 3% *w/v* of orange oil in the film-forming solution. A thinner film resulted in a greater amount of the active compound to migrate from the film.

## 4. Conclusions

In summary, orange oil ME and pectin thin films loaded with orange oil ME were prepared and characterized. Tween 80 as the surfactant and propylene glycol as the co-surfactant at 1:1 ratio were selected to prepare orange oil ME. Three optimized orange oil ME formulations possessed a spherical shape with a droplet size of about 70 nm. Orange oil film (control) showed a loose structure with micropores inside film’s matrix because orange oil evaporated during the drying process. Interestingly, films loaded with orange oil ME exhibited a dense matrix with negligible micropores together with high limonene loading content. Furthermore, the addition of orange oil ME into film improved film flexibility with higher percent elongation and lower Young’s modulus values than the control film. FME 30 film showed higher antimicrobial activity against *S*. *aureus* and *P*. *acnes* than the control film. This study indicated that microemulsion is an interesting technique of potentiating orange oil antibacterial activity in film formulation.

## Figures and Tables

**Table 1:** Compositions of the selected ME formulations and their percent transmittance, particle size, and polydispersity Index (PdI).

Microemulsion	Orange Oil (%)	S_mix_ (%)	Water (%)	Transmittance ± SD (%)	Droplet Size ± SD (nm)	PdI ± SD
ME 20	20	70	10	98.52 ± 0.02 a	73.42 ± 1.60 a	0.216 ± 0.03 a
ME 25	25	65	10	97.51 ± 0.03 b	75.17 ± 7.27 a	0.224 ± 0.03 a
ME 30	30	60	10	96.18 ± 0.01 b	77.63 ± 0.57 a	0.223 ± 0.05 a

For each test, means with the same letter are not significantly different. Thus, means with the different letter, e.g., “a” or “b” are statistically different (*p* < 0.05).

**Table 2:** Mechanical properties and orange oil loading content of films.

Film	Thickness ± SD (mm)	Tensile Strength ± SD (MPa)	Elongation ± SD (%)	Young’s Modulus ± SD (MPa)	Loading Content (%)
Control	0.089 ± 0.009 ^a^	7.28 ± 0.85 ^a^	5.62 ± 0.46 ^a^	129.33 ± 6.83 ^a^	60.75 ± 3.11 ^a^
FME 20	0.094 ± 0.021 ^b^	2.28 ± 0.86 ^b^	7.60 ± 3.01 ^b^	30.52 ± 4.59 ^b^	83.24 ± 5.25 ^b^
FME 25	0.093 ± 0.010 ^b^	3.63 ± 1.36 ^b^	9.29 ± 3.04 ^b^	33.74 ± 3.61 ^b^	83.88 ± 2.43 ^b^
FME 30	0.083 ± 0.017 ^a^	3.24 ± 0.91 ^b^	9.75 ± 0.90 ^b^	34.74 ± 9.01 ^b^	85.10 ± 2.01^b^

**Note**: Control film = film containing orange oil instead of orange oil microemulsion. For each test, means with the same letter are not significantly different. Thus, means with the different letter, e.g., “a” or “b” are statistically different (*p* < 0.05).

**Table 3:** The average diameter (in mm) of inhibition zones of orange oil ME-loaded films (*n* = 5) against the growth of *S. aureus* and *P. acnes.*

Sample	Average Diameter of Inhibition Zones (mm) ± SD	
	*S. aureus*	*P. acnes*
FME 20	ND	13.64 ± 0.25
FME 25	ND	15.18 ± 0.09
FME 30	8.32 ± 0.11	16.10 ± 0.02
Control film	ND	ND
Tween 80	ND	ND
Propylene glycol	ND	ND
Ampicillin (10 mg/mL)	32.87 ± 0.96	19.43 ± 0.60

Note: Control film = film containing orange oil instead of orange oil microemulsion; ND = not detected.
